# *N*-Cyclo­hexyl-3-fluoro­benzamide

**DOI:** 10.1107/S1600536808034478

**Published:** 2008-10-25

**Authors:** Aamer Saeed, Rasheed Ahmad Khera, Naeem Abbas, Ulrich Flörke

**Affiliations:** a Department of Chemistry, Quaid-I-Azam University, Islamabad 45320, Pakistan; b Department Chemie, Fakultät für Naturwissenschaften, Universität Paderborn, Warburgerstrasse 100, D-33098 Paderborn, Germany

## Abstract

In the title mol­ecule, C_13_H_16_FNO, the amide (N—C=O) plane is oriented at an angle of 29.9 (2)° with respect to the aromatic ring. The cyclo­hexane ring adopts the usual chair conformation. In the crystal structure, inter­molecular N—H⋯O hydrogen bonds link the mol­ecules into chains along [100]. A weak C—H⋯F inter­action is also observed. The F atom is disordered over two positions with occupancy factors of 0.873 (3) and 0.127 (3).

## Related literature

For related structures, see: Chopra & Guru Row (2005 ▶); Saeed *et al.* (2008*a* ▶,*b* ▶).
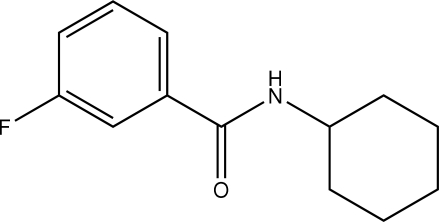


## Experimental

### 

#### Crystal data

C_13_H_16_FNO
*M* *_r_* = 221.27
Monoclinic, 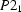

*a* = 5.267 (3) Å
*b* = 6.599 (4) Å
*c* = 16.755 (9) Å
β = 90.090 (17)°
*V* = 582.4 (6) Å^3^
*Z* = 2
Mo *K*α radiation
μ = 0.09 mm^−1^
*T* = 120 (2) K
0.45 × 0.40 × 0.21 mm

#### Data collection

Bruker SMART APEX diffractometer
Absorption correction: multi-scan (*SADABS*; Sheldrick, 2004 ▶) *T*_min_ = 0.962, *T*_max_ = 0.978
5071 measured reflections
1492 independent reflections
1420 reflections with *I* > 2σ(*I*)
*R*_int_ = 0.034

#### Refinement

*R*[*F* ^2^ > 2σ(*F* ^2^)] = 0.035
*wR*(*F* ^2^) = 0.098
*S* = 1.05
1492 reflections
150 parameters
1 restraint
H-atom parameters constrained
Δρ_max_ = 0.25 e Å^−3^
Δρ_min_ = −0.17 e Å^−3^

### 

Data collection: *SMART* (Bruker, 2002 ▶); cell refinement: *SAINT* (Bruker, 2002 ▶); data reduction: *SAINT*; program(s) used to solve structure: *SHELXS97* (Sheldrick, 2008 ▶); program(s) used to refine structure: *SHELXL97* (Sheldrick, 2008 ▶); molecular graphics: *SHELXTL* (Sheldrick, 2008 ▶); software used to prepare material for publication: *SHELXTL*.

## Supplementary Material

Crystal structure: contains datablocks global, I. DOI: 10.1107/S1600536808034478/ci2689sup1.cif

Structure factors: contains datablocks I. DOI: 10.1107/S1600536808034478/ci2689Isup2.hkl

Additional supplementary materials:  crystallographic information; 3D view; checkCIF report

## Figures and Tables

**Table 1:** Hydrogen-bond geometry (Å, °)

*D*—H⋯*A*	*D*—H	H⋯*A*	*D*⋯*A*	*D*—H⋯*A*
N1—H1*A*⋯O1^i^	0.88	2.25	3.050 (2)	152
C5—H5*A*⋯F1^ii^	0.95	2.58	3.310 (3)	134

Symmetry codes: (i) 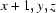; (ii) 
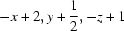
.

**Fig. 1.**
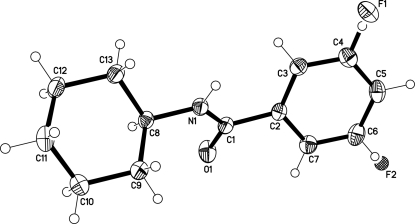
Molecular structure of title compound, showing the rotational disorder of the fluorophenyl ring. Displacement ellipsoids are drawn at the 50% probability level.

**Fig. 2.**
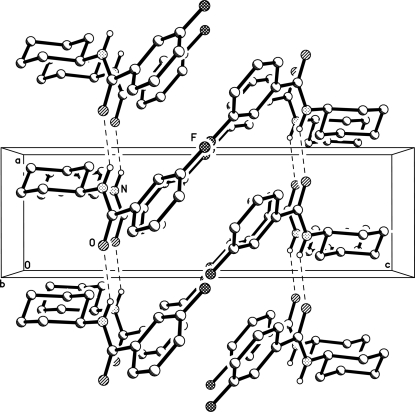
Crystal packing viewed along [100] with intermolecular N–H···O hydrogen bonding pattern indicated as dashed lines. H-atoms not involved in hydrogen bonding are omitted.

## supplementary crystallographic information

### Comment

The background to this study has been described in an earlier paper (Saeed *et al.*, 2008*b*).

The molecular structure of the title compound is related to that of the 2,4-dichloro compound (Saeed *et al.*, 2008*a*). The cyclohexane ring is in the most stable chair conformation. In general, bond lengths and angles are within normal ranges. The aromatic ring C2–C7 is oriented with respect to the N1/O1/C1 plane at a dihedral angle of 29.9 (2)°. The N1–C1–C2–C7 torsion angle is 150.37 (15)°, for the reported dichloro compound the corresponding angle is 130.16 (18)°.

In the crystal structure, intermolecular N—H···O hydrogen bonds (Table 1) link the molecules into infinite chains along the [100] direction (Fig. 2), in which they may be effective in the stabilization of the structure. Another intermolecular interaction is C—H···F (Table 1), as found in 4-fluoro-*N*-(2-fluorophenyl)benzamide (Chopra & Row, 2005).

### Experimental

3,5-Difluorobenzoyl chloride (5.4 mmol) in CHCl_3_ was treated with cyclohexylamine (21.6 mmol) under a nitrogen atmosphere at reflux for 4 h. Upon cooling, the reaction mixture was diluted with CHCl_3_ and washed consecutively with aq 1 *M* HCl and saturated aq NaHCO_3_. The organic layer was dried over anhydrous sodium sulfate and concentrated under reduced pressure. Crystallization of the residue in CHCl_3_ afforded the title compound (84%). Analysis calculated for C_13_H_15_F_2_NO: C 65.26, H 6.32, N 5.85%; found: C 65.31, H 6.39, N 5.77%.

### Refinement

The F atom is disordered over two positions (F1 and F2) with site occupation factors of 0.873 (3) for F1 and 0.127 (3) for F2. H atoms were initially located in difference syntheses, but were then included in the refinement, at calculated positions, in the riding-model approximation, with N—H = 0.88 Å and C—H = 0.95–1.00 Å. The isotropic displacement parameters were set equal to 1.2Ueq of the carrier atom. In the absence of significant anomalous scattering effects, the Friedel pairs were merged.

### Crystal data

**Table:**

C_13_H_16_FNO	*F*(000) = 236
*M**_r_* = 221.27	*D*_x_ = 1.262 Mg m^−^^3^
Monoclinic, *P*2_1_	Mo *K*α radiation, λ = 0.71073 Å
Hall symbol: P 2yb	Cell parameters from 796 reflections
*a* = 5.267 (3) Å	θ = 2.4–28.3°
*b* = 6.599 (4) Å	µ = 0.09 mm^−^^1^
*c* = 16.755 (9) Å	*T* = 120 K
β = 90.090 (17)°	Prism, colourless
*V* = 582.4 (6) Å^3^	0.45 × 0.40 × 0.21 mm
*Z* = 2	

### Data collection

**Table:**

Bruker SMART APEX diffractometer	1492 independent reflections
Radiation source: sealed tube	1420 reflections with *I* > 2σ(*I*)
graphite	*R*_int_ = 0.034
φ and ω scans	θ_max_ = 27.9°, θ_min_ = 2.4°
Absorption correction: multi-scan (SADABS; Sheldrick, 2004)	*h* = −6→6
*T*_min_ = 0.962, *T*_max_ = 0.978	*k* = −8→8
5071 measured reflections	*l* = −22→19

### Refinement

**Table:**

Refinement on *F*^2^	Primary atom site location: structure-invariant direct methods
Least-squares matrix: full	Secondary atom site location: difference Fourier map
*R*[*F*^2^ > 2σ(*F*^2^)] = 0.035	Hydrogen site location: difference Fourier map
*wR*(*F*^2^) = 0.098	H-atom parameters constrained
*S* = 1.05	*w* = 1/[σ^2^(*F*_o_^2^) + (0.0686*P*)^2^ + 0.0394*P*] where *P* = (*F*_o_^2^ + 2*F*_c_^2^)/3
1492 reflections	(Δ/σ)_max_ = 0.001
150 parameters	Δρ_max_ = 0.25 e Å^−^^3^
1 restraint	Δρ_min_ = −0.17 e Å^−^^3^

### Special details

**Table:**

Geometry. All e.s.d.'s (except the e.s.d. in the dihedral angle between two l.s. planes) are estimated using the full covariance matrix. The cell e.s.d.'s are taken into account individually in the estimation of e.s.d.'s in distances, angles and torsion angles; correlations between e.s.d.'s in cell parameters are only used when they are defined by crystal symmetry. An approximate (isotropic) treatment of cell e.s.d.'s is used for estimating e.s.d.'s involving l.s. planes.
Refinement. Refinement of *F*^2^ against ALL reflections. The weighted *R*-factor *wR* and goodness of fit *S* are based on *F*^2^, conventional *R*-factors *R* are based on *F*, with *F* set to zero for negative *F*^2^. The threshold expression of *F*^2^ > σ(*F*^2^) is used only for calculating *R*-factors(gt) *etc*. and is not relevant to the choice of reflections for refinement. *R*-factors based on *F*^2^ are statistically about twice as large as those based on *F*, and *R*- factors based on ALL data will be even larger.

### Fractional atomic coordinates and isotropic or equivalent isotropic displacement parameters (Å^2^)

**Table:**

	*x*	*y*	*z*	*U*_iso_*/*U*_eq_	Occ. (<1)
F1	0.9633 (3)	0.6562 (2)	0.49344 (8)	0.0376 (4)	0.873 (3)
F2	0.249 (2)	0.9837 (17)	0.3846 (6)	0.043 (3)*	0.127 (3)
O1	0.2561 (2)	0.2922 (2)	0.25636 (8)	0.0316 (3)	
N1	0.6841 (3)	0.2208 (2)	0.25218 (8)	0.0206 (3)	
H1A	0.8356	0.2505	0.2710	0.025*	
C1	0.4794 (3)	0.3278 (2)	0.27778 (10)	0.0208 (3)	
C2	0.5341 (3)	0.5002 (2)	0.33504 (9)	0.0198 (3)	
C3	0.7409 (3)	0.4971 (3)	0.38818 (10)	0.0229 (3)	
H3A	0.8556	0.3860	0.3894	0.027*	
C4	0.7720 (3)	0.6615 (3)	0.43877 (10)	0.0269 (4)	
H4A	0.9080	0.6591	0.4760	0.032*	0.127 (3)
C5	0.6128 (4)	0.8300 (3)	0.43745 (10)	0.0288 (4)	
H5A	0.6419	0.9416	0.4721	0.035*	
C6	0.4084 (4)	0.8306 (3)	0.38366 (11)	0.0295 (4)	
H6A	0.2973	0.9439	0.3817	0.035*	0.873 (3)
C7	0.3671 (3)	0.6659 (3)	0.33314 (10)	0.0253 (4)	
H7A	0.2265	0.6658	0.2976	0.030*	
C8	0.6571 (3)	0.0567 (2)	0.19365 (9)	0.0195 (3)	
H8A	0.4853	−0.0054	0.2003	0.023*	
C9	0.6786 (4)	0.1391 (3)	0.10784 (10)	0.0275 (4)	
H9A	0.5431	0.2403	0.0984	0.033*	
H9B	0.8445	0.2074	0.1011	0.033*	
C10	0.6542 (4)	−0.0336 (3)	0.04667 (10)	0.0299 (4)	
H10A	0.4813	−0.0920	0.0497	0.036*	
H10B	0.6781	0.0216	−0.0078	0.036*	
C11	0.8501 (4)	−0.2007 (3)	0.06182 (11)	0.0291 (4)	
H11A	1.0228	−0.1466	0.0524	0.035*	
H11B	0.8212	−0.3136	0.0240	0.035*	
C12	0.8313 (4)	−0.2802 (3)	0.14799 (11)	0.0272 (4)	
H12A	0.6653	−0.3480	0.1555	0.033*	
H12B	0.9664	−0.3818	0.1573	0.033*	
C13	0.8582 (3)	−0.1078 (3)	0.20882 (11)	0.0232 (3)	
H13A	1.0299	−0.0476	0.2047	0.028*	
H13B	0.8379	−0.1624	0.2635	0.028*	

### Atomic displacement parameters (Å^2^)

**Table:**

	*U*^11^	*U*^22^	*U*^33^	*U*^12^	*U*^13^	*U*^23^
F1	0.0405 (8)	0.0402 (7)	0.0322 (7)	−0.0017 (6)	−0.0107 (5)	−0.0067 (6)
O1	0.0177 (6)	0.0310 (7)	0.0460 (8)	0.0009 (5)	−0.0028 (5)	−0.0113 (6)
N1	0.0172 (6)	0.0198 (6)	0.0247 (7)	0.0003 (5)	−0.0015 (5)	−0.0024 (5)
C1	0.0197 (8)	0.0181 (7)	0.0246 (7)	0.0002 (6)	0.0004 (6)	−0.0004 (6)
C2	0.0197 (8)	0.0180 (7)	0.0217 (7)	−0.0015 (6)	0.0045 (6)	0.0002 (6)
C3	0.0231 (8)	0.0221 (7)	0.0235 (7)	0.0014 (6)	0.0018 (6)	0.0010 (6)
C4	0.0270 (9)	0.0306 (9)	0.0229 (8)	−0.0040 (7)	0.0018 (6)	−0.0012 (7)
C5	0.0314 (9)	0.0259 (9)	0.0292 (8)	−0.0041 (7)	0.0075 (7)	−0.0081 (8)
C6	0.0268 (9)	0.0233 (8)	0.0385 (9)	0.0044 (7)	0.0076 (7)	−0.0052 (8)
C7	0.0214 (8)	0.0253 (8)	0.0293 (8)	0.0023 (7)	0.0013 (6)	−0.0015 (7)
C8	0.0169 (7)	0.0173 (7)	0.0243 (8)	−0.0005 (6)	−0.0003 (6)	−0.0017 (6)
C9	0.0376 (10)	0.0205 (8)	0.0242 (8)	0.0018 (7)	−0.0026 (7)	0.0000 (6)
C10	0.0367 (10)	0.0281 (9)	0.0250 (8)	−0.0007 (8)	−0.0034 (7)	−0.0043 (7)
C11	0.0290 (9)	0.0249 (8)	0.0334 (9)	−0.0032 (8)	0.0051 (7)	−0.0085 (8)
C12	0.0284 (9)	0.0177 (8)	0.0356 (9)	0.0023 (7)	−0.0016 (7)	−0.0027 (7)
C13	0.0221 (8)	0.0192 (8)	0.0285 (8)	0.0014 (6)	−0.0010 (6)	−0.0003 (6)

### Geometric parameters (Å, °)

**Table:**

F1—C4	1.361 (2)	C8—C13	1.538 (2)
F2—C6	1.315 (11)	C8—C9	1.541 (2)
O1—C1	1.252 (2)	C8—H8A	1.00
N1—C1	1.359 (2)	C9—C10	1.538 (2)
N1—C8	1.468 (2)	C9—H9A	0.99
N1—H1A	0.88	C9—H9B	0.99
C1—C2	1.515 (2)	C10—C11	1.531 (3)
C2—C7	1.404 (2)	C10—H10A	0.99
C2—C3	1.406 (2)	C10—H10B	0.99
C3—C4	1.386 (3)	C11—C12	1.540 (3)
C3—H3A	0.95	C11—H11A	0.99
C4—C5	1.393 (3)	C11—H11B	0.99
C4—H4A	0.95	C12—C13	1.534 (2)
C5—C6	1.403 (3)	C12—H12A	0.99
C5—H5A	0.95	C12—H12B	0.99
C6—C7	1.394 (3)	C13—H13A	0.99
C6—H6A	0.95	C13—H13B	0.99
C7—H7A	0.95		
			
C1—N1—C8	121.23 (14)	C13—C8—H8A	108.4
C1—N1—H1A	119.4	C9—C8—H8A	108.4
C8—N1—H1A	119.4	C10—C9—C8	110.76 (15)
O1—C1—N1	123.89 (15)	C10—C9—H9A	109.5
O1—C1—C2	120.02 (14)	C8—C9—H9A	109.5
N1—C1—C2	116.09 (14)	C10—C9—H9B	109.5
C7—C2—C3	120.72 (15)	C8—C9—H9B	109.5
C7—C2—C1	116.85 (14)	H9A—C9—H9B	108.1
C3—C2—C1	122.42 (15)	C11—C10—C9	111.55 (14)
C4—C3—C2	117.78 (16)	C11—C10—H10A	109.3
C4—C3—H3A	121.1	C9—C10—H10A	109.3
C2—C3—H3A	121.1	C11—C10—H10B	109.3
F1—C4—C3	118.55 (17)	C9—C10—H10B	109.3
F1—C4—C5	118.43 (16)	H10A—C10—H10B	108.0
C3—C4—C5	123.00 (16)	C10—C11—C12	110.90 (15)
C3—C4—H4A	118.5	C10—C11—H11A	109.5
C5—C4—H4A	118.5	C12—C11—H11A	109.5
C4—C5—C6	118.30 (16)	C10—C11—H11B	109.5
C4—C5—H5A	120.9	C12—C11—H11B	109.5
C6—C5—H5A	120.9	H11A—C11—H11B	108.0
F2—C6—C7	120.5 (5)	C13—C12—C11	111.33 (15)
F2—C6—C5	119.0 (5)	C13—C12—H12A	109.4
C7—C6—C5	120.42 (16)	C11—C12—H12A	109.4
C7—C6—H6A	119.8	C13—C12—H12B	109.4
C5—C6—H6A	119.8	C11—C12—H12B	109.4
C6—C7—C2	119.75 (15)	H12A—C12—H12B	108.0
C6—C7—H7A	120.1	C12—C13—C8	110.55 (13)
C2—C7—H7A	120.1	C12—C13—H13A	109.5
N1—C8—C13	110.16 (13)	C8—C13—H13A	109.5
N1—C8—C9	110.86 (13)	C12—C13—H13B	109.5
C13—C8—C9	110.58 (13)	C8—C13—H13B	109.5
N1—C8—H8A	108.4	H13A—C13—H13B	108.1
			
C8—N1—C1—O1	2.5 (2)	F2—C6—C7—C2	−177.6 (5)
C8—N1—C1—C2	−177.01 (13)	C5—C6—C7—C2	−1.2 (3)
O1—C1—C2—C7	−29.1 (2)	C3—C2—C7—C6	0.9 (2)
N1—C1—C2—C7	150.37 (15)	C1—C2—C7—C6	−179.27 (15)
O1—C1—C2—C3	150.71 (16)	C1—N1—C8—C13	−148.28 (15)
N1—C1—C2—C3	−29.8 (2)	C1—N1—C8—C9	89.00 (18)
C7—C2—C3—C4	0.6 (2)	N1—C8—C9—C10	179.17 (14)
C1—C2—C3—C4	−179.20 (14)	C13—C8—C9—C10	56.69 (18)
C2—C3—C4—F1	176.62 (15)	C8—C9—C10—C11	−55.8 (2)
C2—C3—C4—C5	−1.9 (3)	C9—C10—C11—C12	55.0 (2)
F1—C4—C5—C6	−176.95 (16)	C10—C11—C12—C13	−55.56 (19)
C3—C4—C5—C6	1.6 (3)	C11—C12—C13—C8	56.76 (19)
C4—C5—C6—F2	176.4 (6)	N1—C8—C13—C12	179.90 (13)
C4—C5—C6—C7	0.1 (3)	C9—C8—C13—C12	−57.21 (18)

### Hydrogen-bond geometry (Å, °)

**Table:**

*D*—H···*A*	*D*—H	H···*A*	*D*···*A*	*D*—H···*A*
N1—H1A···O1^i^	0.88	2.25	3.050 (2)	152
C5—H5A···F1^ii^	0.95	2.58	3.310 (3)	134

Symmetry codes: (i) *x*+1, *y*, *z*; (ii) −*x*+2, *y*+1/2, −*z*+1.
